# The EH domain-containing protein, EdeA, is involved in endocytosis, cell wall integrity, and pathogenicity in *Aspergillus fumigatus*

**DOI:** 10.1128/msphere.00057-24

**Published:** 2024-04-30

**Authors:** Mengyao Dai, Xintian Liu, Gustavo H. Goldman, Ling Lu, Shizhu Zhang

**Affiliations:** 1 Jiangsu Key Laboratory for Microbes and Functional Genomics, Jiangsu Engineering and Technology Research Centre for Microbiology, College of Life Sciences, Nanjing Normal University, Nanjing, China; 2 Faculdade de Ciências Farmacêuticas de Ribeirão Preto, Universidade de São Paulo, Ribeirão Preto, Brazil; CNRS-INSERM-Université Côte d'Azur, Nice, France

**Keywords:** *Aspergillus fumigatus*, endocytosis, EdeA, EH domains, cell wall integrity

## Abstract

Endocytosis has been extensively studied in yeasts, where it plays crucial roles in growth, signaling regulation, and cell-surface receptor internalization. However, the biological functions of endocytosis in pathogenic filamentous fungi remain largely unexplored. In this study, we aimed to functionally characterize the roles of EdeA, an ortholog of the *Saccharomyces cerevisiae* endocytic protein Ede1, in *Aspergillus fumigatus*. EdeA was observed to be distributed as patches on the plasma membrane and concentrated in the subapical collar of hyphae, a localization characteristic of endocytic proteins. Loss of *edeA* caused defective hyphal polarity, reduced conidial production, and fewer sites of endocytosis initiations than that of the parental wild type. Notably, the *edeA* null mutant exhibited increased sensitivity to cell wall-disrupting agents, indicating a role for EdeA in maintaining cell wall integrity in *A. fumigatus*. This observation was further supported by the evidence showing that the thickness of the cell wall in the Δ*edeA* mutant increased, accompanied by abnormal activation of MpkA, a key component in the cell wall integrity pathway. Additionally, the Δ*edeA* mutant displayed increased pathogenicity in the *Galleria mellonella* wax moth infection model, possibly due to alterations in cell wall morphology. Site-directed mutagenesis identified the conserved residue E348 within the third EH (Eps15 homology) domain of EdeA as crucial for its subcellular localization and functions. In conclusion, our results highlight the involvement of EdeA in endocytosis, hyphal polarity, cell wall integrity, and pathogenicity in *A. fumigatus*.

**IMPORTANCE:**

*Aspergillus fumigatus* is a significant human pathogenic fungus known to cause invasive aspergillosis, a disease with a high mortality rate. Understanding the basic principles of *A. fumigatus* pathogenicity is crucial for developing effective strategies against this pathogen. Previous research has underscored the importance of endocytosis in the infection capacity of pathogenic yeasts; however, its biological function in pathogenic mold remains largely unexplored. Our characterization of EdeA in *A. fumigatus* sheds light on the role of endocytosis in the development, stress response, and pathogenicity of pathogenic molds. These findings suggest that the components of the endocytosis process may serve as potential targets for antifungal therapy.

## INTRODUCTION

Endocytosis is a fundamental cellular process in eukaryotic cells that facilitates the transport of molecules into cells. Previous studies in *Saccharomyces cerevisiae* have highlighted the role of endocytosis in the internalization of the α-factor receptor via the endocytic proteins END3 and END4 (1). In *Candida albicans*, disruption of *end3* affects cell wall integrity and alters susceptibility to azole drugs. Meanwhile, the secretion of the proteases Saps was reduced in the *end3* null mutant, leading to a decrease in adhesion and hence pathogenicity (2). Additionally, the absence of the endocytic vesicle division proteins amphiphysin *rvs161* and *rvs167* in *C. albicans* affects hyphal growth and the occurrence of oropharyngeal candidiasis (3). In *Cryptococcus neoformans*, several components of clathrin-mediated endocytosis including Chc1, Las17, Rvs161, and Rvs167 are required for the internalization of heme and hemoglobin, with a *las17* mutant displaying avirulent in a Cryptococcosis mouse model (4).

While endocytosis has been extensively studied in yeast, recent research has emphasized its crucial roles in filamentous fungi development. Filamentous fungi exhibit rapid growth through apical extensions, forming elongated tubular cells known as hyphae (5). Research on hyphal growth has traditionally focused on exocytosis mediated by the Spitzenkörper, a vesicle supply center that coordinates vesicle delivery to the plasma membrane (6, 7). Studies in model filamentous fungi such as *Aspergillus nidulans* and *Neurospora crassa* have highlighted the importance of the subapical collar, rich in endocytosis, for hyphal growth (8–10). Endocytosis is concentrated at the endocytic collar surrounding the cell tip, which may counteract passive protein diffusion from the growth site (11, 12). Inhibition of endocytosis disrupts polarity, alters cell shape, and severely impairs growth (13, 14). Given that the polarized growth of filamentous fungi aids in substrate exploration and tissue invasion, the endocytic machinery is likely to play a critical role in their pathogenicity.

*Aspergillus fumigatus*, a successful pathogen in immunocompromised patients (15, 16), can cause various human diseases, with invasive aspergillosis (IA) being the most severe (17). The high mortality rate associated with IA has prompted extensive efforts to elucidate the fundamental principles underlying the pathogenicity of *A. fumigatus*. The specific impact of endocytosis on the growth, development, and pathogenicity of *A. fumigatus* remains poorly understood.

Ede1, an orthologue of mammalian Eps15, serves as a key early protein in clathrin-mediated endocytosis in *S. cerevisiae*, facilitating the targeting of endocytic sites (18–20). Deletion of *ede1* leads to a reduction in clathrin-mediated endocytosis initiations, disruptions in vesicle maturation timing, and a decrease in the lifetimes of other endocytic proteins (19, 21). While the deletion of *ede1* is not essential, it does impact the diploid budding pattern (22). Conversely, deletion of *Caede1*, the *ede1* ortholog in the human fungal pathogen *C. albicans*, does not exhibit notable defects during growth in yeast or hyphal stages and only minimally affects the budding pattern (23). Structurally, Ede1 contains three Eps15 homology (EH) domains at its N terminus, a coiled-coiled region, and a ubiquitin-binding associated (UBA) domain at its C terminus (21, 24, 25). Studies have suggested that the EH domains within Ede1 play crucial roles in its interactions with partners such as the AP-2 complex, casein kinase 1, and Syp1 (26, 27). However, the specific amino acid sites within the EH domains that are crucial for its functionality remain subjects of ongoing investigation.

In our current study, we have identified EdeA, the *A. fumigatus* ortholog of *S. cerevisiae* Ede1, as playing significant roles in endocytosis, hyphal growth, cell wall integrity, and the normal pathogenicity of *A. fumigatus*. Additionally, we have demonstrated that the conserved residue E348 within the third EH domain plays a critical role in the subcellular localization and functional performance of EdeA. Collectively, our findings underscore the involvement of the endocytic protein EdeA in hyphal growth, cell wall integrity, and pathogenicity in *A. fumigatus*.

## RESULTS

### Phylogenetic analysis of yeast Ede1 orthologs and their conserved domains

To identify the ortholog of Ede1 in *A. fumigatus*, the amino acid sequence of Ede1 from the budding yeast *S. cerevisiae* was utilized as a query in a BLASTp search against the *A. fumigatus* genome. In this way, a calcium ion binding protein AFUB_002920 (*E* value, 8*e*^−51^ and identity, 31.16%) was found to be the best match. Simultaneously, a reverse comparison was performed using the amino acid sequence of AFUB_002920 in *A. fumigatus* as a query to search the *S. cerevisiae* genome for the orthologs. As expected, Ede1 was the optimal match (*E* value, 3*e*^−53^ and identity, 30.9%). These findings indicate that the ortholog of Ede1 in *A. fumigatus* is AFUB_002920, designated as EdeA.

**Fig 1:**
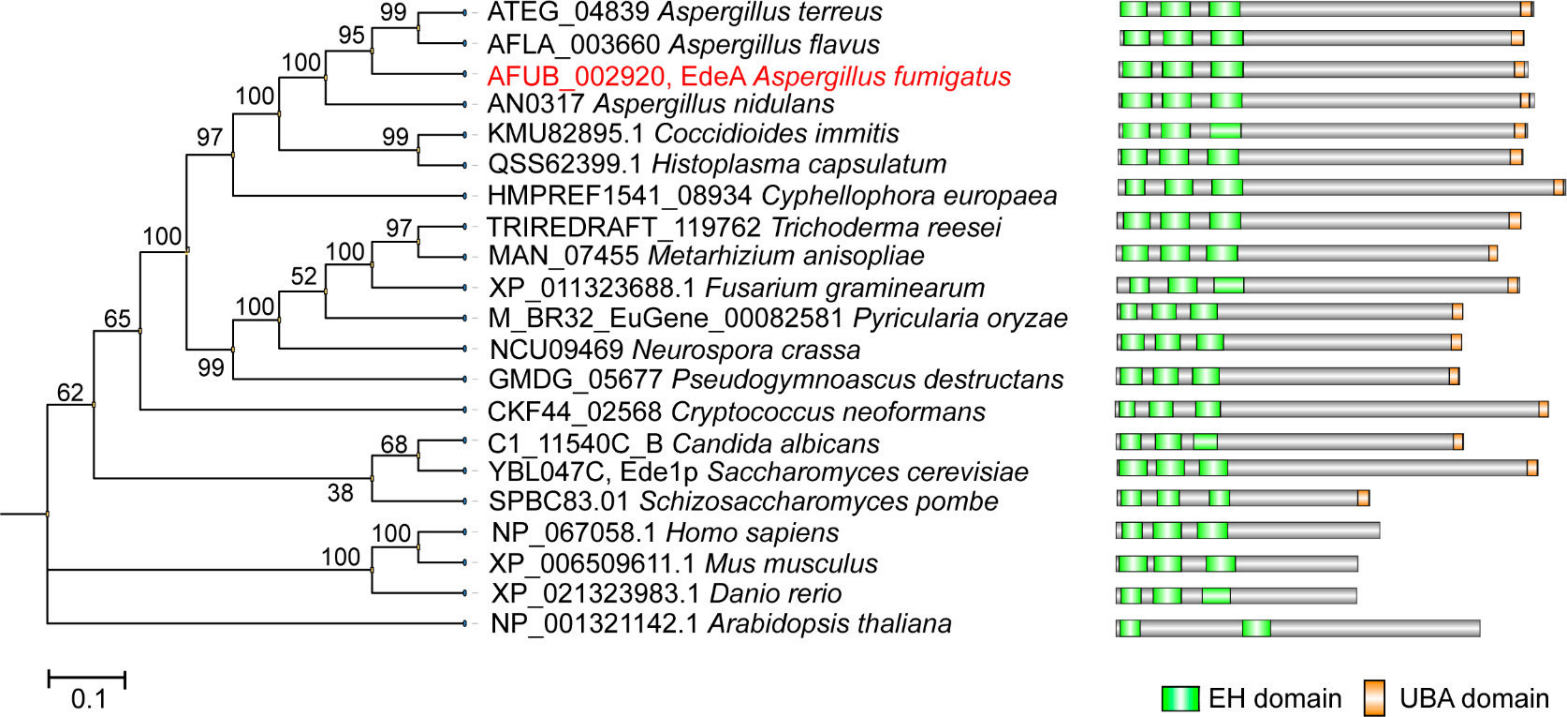
Phylogenetic relationship and protein domain analysis of Ede1 orthologs in selected species. The MEGA11 software was used to conduct phylogenetic tree analysis of selected genes. The phylogenetic tree was constructed using the neighbor-joining method based on the full length of the Ede1 ortholog proteins, with the number of bootstrap replicates set to 1,000. Protein domain analysis was performed using the conserved domain search function on the NCBI website, and the protein domains were visualized using IBS software.

The *edeA* gene spans 3,777 bp and encodes a protein composed of 1,258 amino acids. Phylogenetic analysis further reveals that EdeA is evolutionarily conserved from fungi to mammals (Fig. 1). It is noteworthy that, with the exception of *Arabidopsis thaliana*, which contains two EH domains, all selected Ede1/EdeA orthologs feature three conserved EH domains, suggesting the critical importance of this domain for their functions. Additionally, a search using the conserved domain search tool on the NCBI website revealed that the UBA domain is uniquely present in fungi and absent in mammals and plants.

### Loss of *edeA* affects the hyphal growth, asexual development, and polarity of *A. fumigatus*

**Fig 2:**
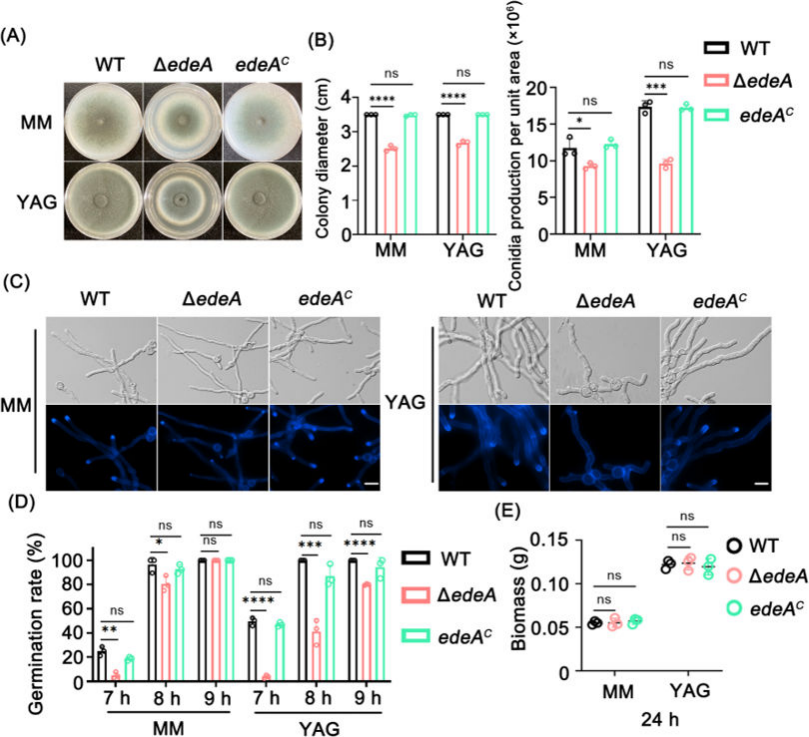
The loss of *edeA* affects the hyphal growth and asexual development of *A. fumigatus*. (**A**) Growth phenotypes of the indicated strains on solid MM and YAG media at 37°C for 72 h. (**B**) Quantitative analysis of colony diameter and conidia production per unit area. (**C**) Growth phenotypes of the indicated strains were observed after static growth in liquid MM and YG media at 37°C for 13 h. Calcofluor white was used to detect the presence of chitin. Scale bar: 10 µm. (**D**) Quantitative analysis of WT, Δ*edeA*, and *edeA^C^* germination rate in liquid MM and YG media after 7 h, 8 h, and 9 h static growth. (**E**) Quantitative analysis of WT, Δ*edeA*, and *edeA^C^* biomass in liquid MM and YG media after shaking culture for 24 h. Statistical analysis was performed using one-tailed, unpaired *t*-tests. ns, not significant; *P < 0.05, **P < 0.01, ***P < 0.001, and ****P < 0.0001.

The *edeA* deletion strain was generated through homologous gene replacement using *pyr4* as a marker (Fig. S1). To explore the function of the *edeA* gene in the development of *A. fumigatus*, conidia of the corresponding strains were spot inoculated on solid MM (minimal medium) and YAG (yeast extract glucose) medium. As shown in Fig. 2A and B, the Δ*edeA* mutant exhibited a significant reduction in colony growth on both MM (23.53% reduction) and YAG (28.19% reduction) media compared with the wild type (WT). Furthermore, conidial production was lower in the Δ*edeA* mutant when grown on MM (20.88% reduction) and YAG (44.5% reduction) media, indicating a role for *edeA* in the asexual development of *A. fumigatus*.

Interestingly, the absence of *edeA* led to decreased growth and an abnormal hyphal morphology characterized by an increased number of branching sites compared with the parental WT strain when cultured in submerged liquid YG medium, suggesting a role for *edeA* in hyphal polarity. Additionally, the apical chitin contents, as stained by calcofluor white (CFW), were significantly decreased in the Δ*edeA* mutant in both liquid MM and YG media (Fig. 2C). Loss of *edeA* resulted in a delay in germination but did not affect the 24-h biomass (Fig. 2D and E). The complemented strain *edeA^C^*, which ectopically expressed *edeA* in the Δ*edeA* mutant, exhibited a phenotype almost identical to that of the WT strain, indicating that these observed phenotypes are specifically attributed to the loss of *edeA*. Overall, these findings suggest that *edeA* plays a crucial role in hyphal growth, asexual development, and hyphal polarity in *A. fumigatus*.

### EdeA localizes at the subapical collar of hyphae in *A. fumigatus*

**Fig 3:**
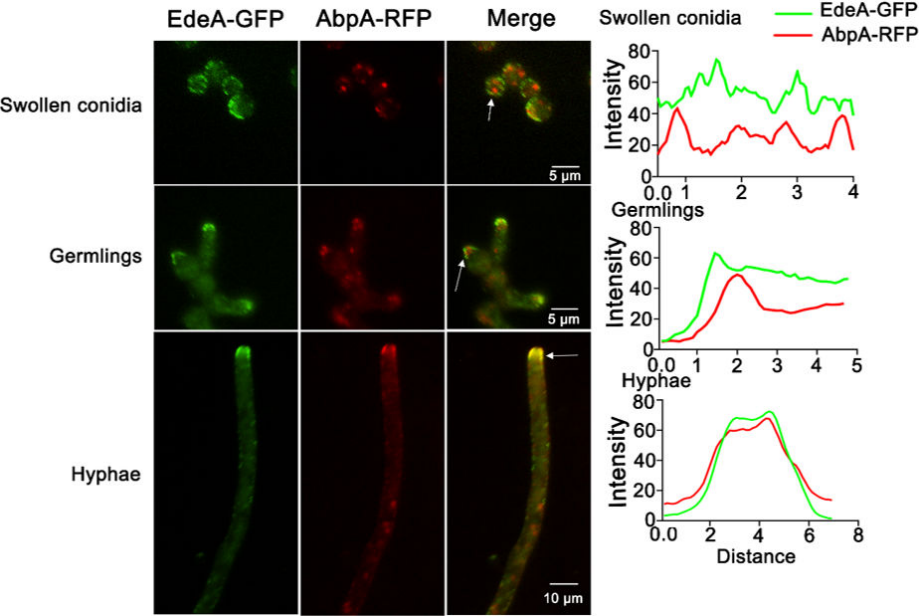
Subcellular localization of EdeA and AbpA during developmental stages in *A. fumigatus*. Fresh conidia of the EdeA-GFP and AbpA-RFP colocalization strain were collected and cultured in liquid MM for 6 h (swollen conidia), 8 h (germlings), and 13 h (hyphae). Right panel: intensity quantification distributions of EdeA-GFP and AbpA-RFP were analyzed to show co-localized content relative to the left image using ImageJ software. Arrows point to position of analysis.

To observe the localization of EdeA, we constructed a C-terminally labeled GFP strain of EdeA under the control of the native promoter. The growth of the EdeA-GFP strain was identical to that of the WT, indicating that the addition of the GFP tag did not affect the function of EdeA (Fig. S2). In swollen conidia, EdeA-GFP was distributed evenly throughout the cell and appeared non-polarized. In germlings, EdeA-GFP was concentrated at the tip of the germ tube. In the mature hyphae, EdeA-GFP was distributed as patches on the plasma membrane and concentrated in the subapical collar of the hyphae, a characteristic location of endocytic proteins (Fig. 3). To further confirm the localization of EdeA, AbpA, a marker of endocytic internalization, was labeled with RFP (28). As expected, EdeA-GFP mainly colocalizes with AbpA-RFP in the subapical collar of hyphal cells. No obvious colocalization between EdeA-GFP and AbpA-RFP was observed during the swollen conidia and germling stages (Fig. 3).

### EdeA contributes to the initiation of endocytosis of *A. fumigatus*

A conserved function of the EdeA orthologs Ede1/EPS15 in *S. cerevisiae* and mammals was in the process of endocytosis (25, 29). To investigate whether EdeA is also involved in endocytosis in *A. fumigatus*, membrane internalization was observed using FM4-64 staining. FM4-64 is a membrane-selective fluorescent vital dye that has been utilized as a marker of endocytosis in live cells (30).

**Fig 4:**
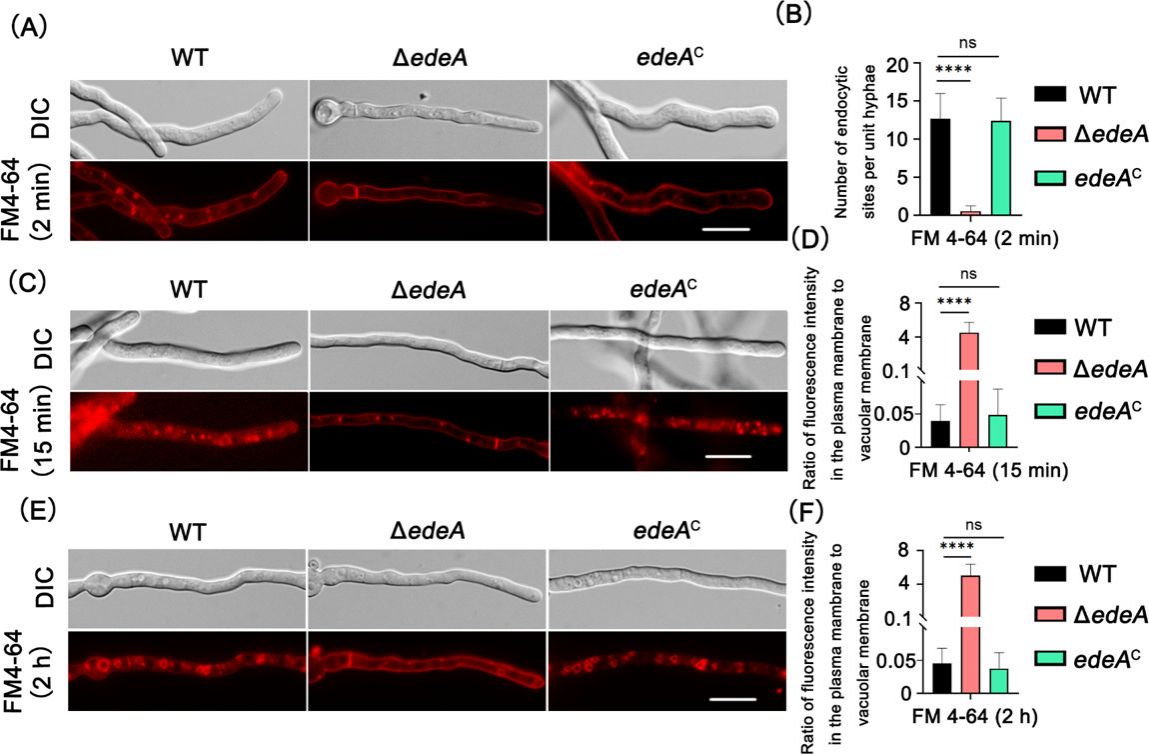
EdeA affects the formation of endocytic internalization initiation sites in *A. fumigatus*. (**A**) Fluorescence distribution of hyphal cells stained with FM4-64. Fresh conidia of the indicated strains were cultivated in liquid MM medium for 13 h and subsequently stained with FM4-64 for 2 min on ice. (**B**) Quantitative analysis of the number of endocytosis sites on the plasma membrane after 2 min of staining with FM4-64. (**C**) Distribution of endosomes/vacuoles in hyphal cells stained with FM4-64. Fresh conidia of the indicated strains were grown in liquid MM medium for 13 h and then stained with FM4-64 for 15 min at room temperature. (**D**) Graph showing the ratio of fluorescence intensity in the plasma membrane to the vacuolar membrane stained with the indicated strains as shown in panel C. (**E**) Distribution of larger vacuoles in hyphal cells stained with FM4-64. Fresh conidia of the indicated strains were grown in liquid MM medium for 13 h and then stained with FM4-64 for 2 h at room temperature. (**F**) Graph showing the ratio of fluorescence intensity in the plasma membrane to the vacuolar membrane of the indicated strains as shown in panel E. Statistical analysis was performed using one-tailed, unpaired *t*-tests. ns, not significant; ****P < 0.0001. Scale bar: 10 µm.

The plasma membrane was stained within 2 min, delineating the typical shape of the hyphae and forming small endocytic sites in both the WT strain and the complemented strain, as depicted in Fig. 4A and B. At this initial time point, the *edeA* null mutant can be only vaguely seen for the straining shape of the hyphae without cortical patches forming. After 15 min of staining, the accumulation of FM4-64 in mature endosomes/vacuoles was observed in both the WT strain and the complemented strain, indicating the entry of FM4-64 primarily through endocytic vesicles invaginated from the plasma membrane, as shown in Fig. 4C and D. In contrast, the Δ*edeA* mutant displayed a notable delay in the endocytosis process, with only a limited number of endocytosis sites observed after the same 15-min staining period (Fig. 4C and D). Subsequent to extending the staining time by 2 h, conspicuous large-volume vacuoles were observed in the WT and complemented strains (Fig. 4E and F). At this later time point, the *edeA* null mutant also developed vacuoles, but in significantly fewer numbers and with less distinct shapes compared with the WT strain. Overall, the findings indicate that the loss of *edeA* led to a substantial reduction in endocytosis initiation sites without complete inhibition of the endocytosis process.

### The EdeA contributes to cell wall integrity in *A. fumigatus*

**Fig 5:**
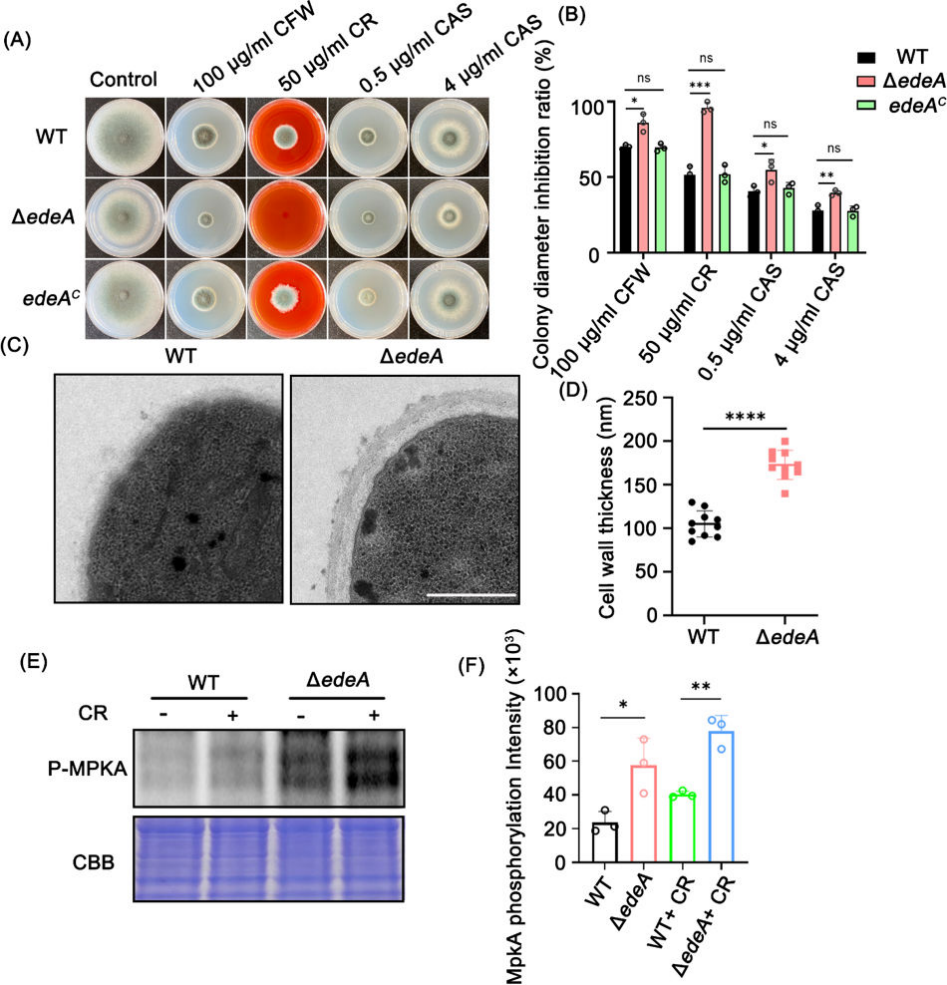
EdeA is involved in cell wall integrity in *A. fumigatus*. (**A**) Colony growth of related strains grown on MM in the presence and absence of cell wall-disrupting reagents CFW, CR, and CAS at 37°C for 72 h. (**B**) Relative hyphal growth inhibition of the indicated strains as shown in panel A. Data are presented as mean ± SD from three independent experiments. (**C**) Representative TEM images of WT and the *edeA* null mutant hyphae cultured in MM. Scale bar: 1 µm. (**D**) Quantification of the mean cell wall thickness of WT and the *edeA* null mutant as shown in panel C. The data are presented as the mean ± SD of three biological samples, with 10 sections measured for each. (**E**) Phosphorylation levels of MpkA in WT and *edeA* null mutant in the presence and absence of CR. The indicated strains were grown in liquid MM for 23 h, and then, CR at a final concentration of 300 µg/mL was added for further 1 h. Coomassie Brilliant Blue (CBB) gel of the protein extract was used as a loading control. (**F**) The bar graph quantifies the signal intensity of MpkA phosphorylation levels of the strains shown. Statistical analysis was performed using one-tailed, unpaired *t*-tests. ns, not significant; *P < 0.05, **P < 0.01, ***P < 0.001, and ****P < 0.0001.

Endocytosis is crucial for hyphal growth and the polarized localization of cell wall-modifying enzymes (14, 31). To further determine whether EdeA is involved in the cell wall integrity of *A. fumigatus*, we inoculated the indicated strains onto plates containing various cell wall-disrupting reagents, including Calcofluor white (CFW), Congo red (CR), and Caspofungin (CAS). The results showed that the Δ*edeA* mutant was more sensitive to all the tested cell wall-disrupting reagents to varying degrees compared with the WT and the complemented strains (Fig. 5A and B). Among them, the Δ*edeA* mutant proved to be the most sensitive to CR, inhibiting almost 100% of the colony diameter compared with 50% inhibition in the WT. In addition, the thickness of the Δ*edeA* mutant cell wall inspected by transmission electron microscopy (TEM) was much thicker than that in the WT strain (Fig. 5C and D), indicating that EdeA plays a role in cell wall architecture.

The cell wall integrity (CWI) signaling pathway regulates the shape and biosynthesis of the cell wall (32). MpkA is a key component of the CWI pathway; it is phosphorylated under cell wall and other stresses (33). To test whether EdeA is involved in the CWI signaling pathway, the levels of MpkA phosphorylation in both the WT and Δ*edeA* mutant were assayed. CR significantly induced the phosphorylation of MpkA in both the WT and the Δ*edeA* mutant. However, the phosphorylation of MpkA was much higher in the Δ*edeA* mutant compared with the WT both in the presence and absence of CR (Fig. 5E and F). In particular, the phosphorylation of MpkA in the Δ*edeA* mutant was abnormally induced in the absence of CR, indicating that the cell wall integrity of Δ*edeA* is indeed compromised. Taken together, these findings suggest that EdeA is involved in cell wall integrity in *A. fumigatus*.

### A conserved glutamate within the EH domain is important for the subcellular localization and function of EdeA

**Fig 6:**
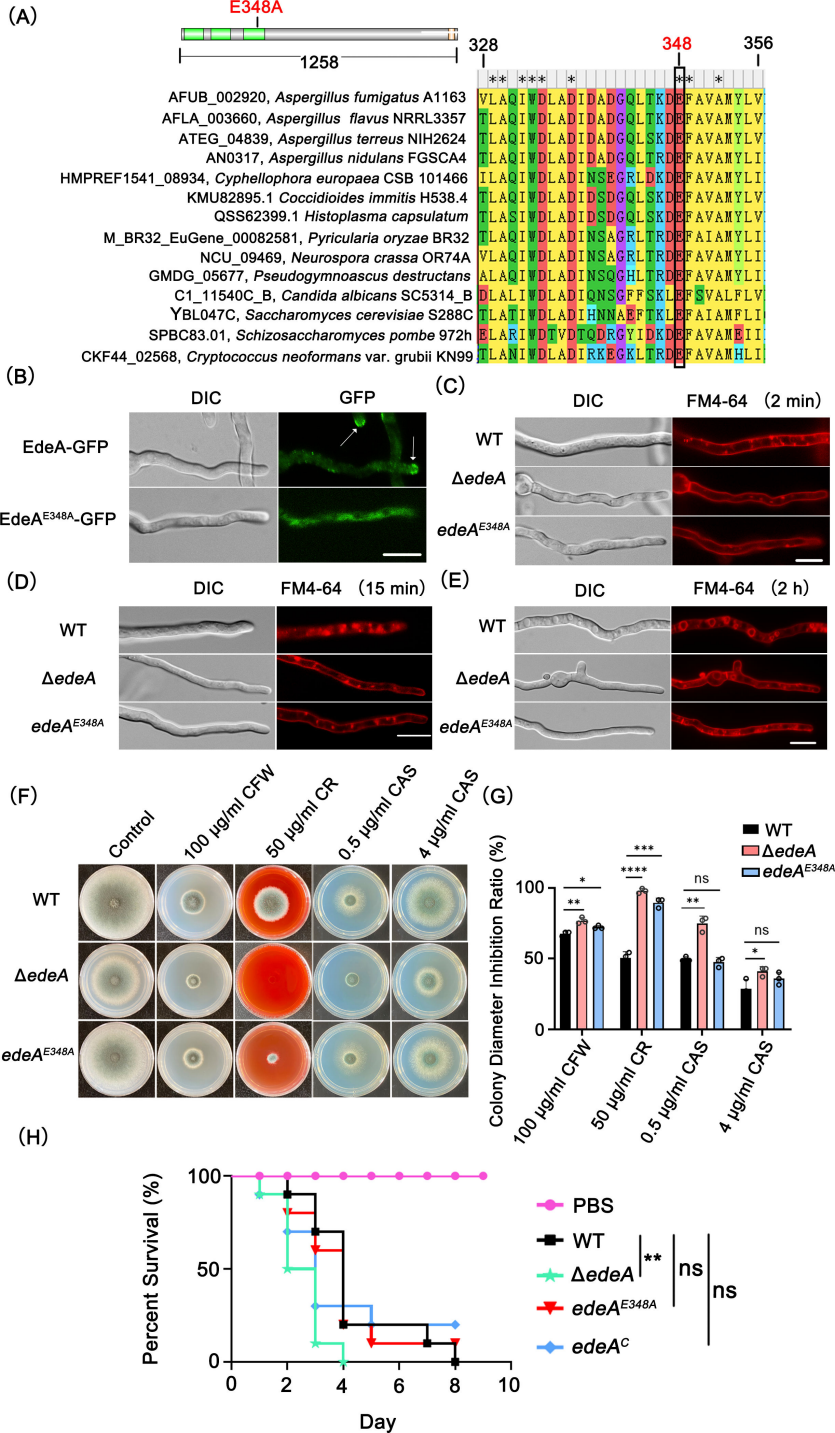
The E348 residue in the EH domain plays a critical role in the subcellular localization and function of EdeA. (**A**) Comparison of the amino acid sequences of the EH domains of EdeA orthologs in selected fungal species using Clustal W. Highly conserved residues are shown in red letters, while the black box indicates sites with the ability to bind calcium ions. (**B**) Subcellular localization of EdeA-GFP and EdeA^E348A^-GFP in hyphae. The specified stains were cultured in liquid MM for 13 h at 37°C. Arrows indicate the subapical collar localization of EdeA. (**C–E**) The fluorescence distribution of hyphal cells stained with FM4-64. Fresh conidia of WT, Δ*edeA*, and *edeA^E348A^* strains were cultivated in liquid MM medium for 13 h and subsequently stained with FM4-64 for 2 min, 15 min, and 2 h, respectively. (**F**) Colony growth of related strains was observed on MM in the presence and absence of CFW, CR, and CAS condition at 37°C for 72 h. (**G**) Relative hyphal growth inhibition of the indicated strains as shown in panel F. Data are shown as mean ± SD from three independent experiments. Statistical analysis was performed using one-tailed, unpaired *t*-tests. ns, not significant; *P < 0.05, **P < 0.01, ***P < 0.001, and ****P < 0.0001. Scale bar: 10 µm. (**H**) Survival curves for *G. mellonella* larvae infected with the WT, Δ*edeA*, *edeA^E348A^*, and *edeA^C^* strains. PBS-injected larvae were used as a negative control. Statistical analysis between groups were conducted using the log-rank test. ns, not significant; **P < 0.01.

EH domains are crucial for the function of Ede1 by interacting with its partners (34). Interestingly, each EH domain contains a pair of EF-hand motifs, which have the ability to bind calcium ions (35). EdeA contains three conserved EH domains, and the amino acid sequence alignment analysis showed that glutamate (E348) in the third EF-hand domain, which is predicted to have the ability to bind calcium, is conserved in all the selected fungi (Fig. 6A). To investigate whether the conserved glutamate within the third EH domain is essential for the subcellular localization and function of EdeA, a site-directed mutant (E348A) was created. In this mutant, the glutamate was substituted with alanine. The mutant strains, EdeA^E348A^ and EdeA^E348A^-GFP, were generated in both the WT and EdeA-GFP backgrounds, respectively.

As mentioned above, the EdeA-GFP protein was concentrated in a sub-apical collar of the hyphae. In comparison, EdeA^E348A^-GFP displays a smeared-out boundary distribution, with the loss of subapical collar localization (Fig. 6B). Furthermore, EdeA^E348A^ showed similarly fewer endocytosis initiation sites as the Δ*edeA* mutant than its wild type, suggesting that this site E348 in EdeA in *A. fumigatus* plays a critical role in the endocytosis process (Fig. 6C through E). However, the growth assay showed that there was no significant difference in the colony growth between the EdeA^E348A^ mutant and WT when cultured on MM, indicating that the E348 site has a minor effect on the radial growth of *A. fumigatus* (Fig. 6F and G). To further determine the role of this site in cell wall integrity, we inoculated the indicated strains on MM and MM plus cell wall-disrupting reagents. The EdeA^E348A^ mutant showed an increased sensitive phenotype to CR and CFW, but to a lesser extent than the Δ*edeA* mutant (Fig. 6F and G).

We further investigated whether the EdeA and E348 sites contribute to the pathogenicity of *A. fumigatus* by using the *G. mellonella* wax moth infection model (36). Survival analysis showed that the Δ*edeA* mutant but not the EdeA^E348A^ mutant exhibited a significantly increased mortality rate (*P* < 0.01) of larvae compared with the WT and complemented strains (Fig. 6H). The median survival times of the WT, Δ*edeA*, *edeA^E348A^*, and *edeA^C^* are 4 days, 2.5 days, 4 days, and 4 days, respectively. In summary, the results suggest that the E348 residue of EdeA is involved in subcellular localization and functions such as endocytosis and cell wall integrity, but not in radial growth and pathogenicity.

## DISCUSSION

This study identified EdeA, an ortholog of Ede1 in the budding yeast *S. cerevisiae*, which is involved in endocytosis, hyphal polarity, cell wall integrity, and pathogenicity in *A. fumigatus*. EdeA-GFP was concentrated in the subapical collar of hyphae, a location characteristic of endocytic proteins. This observation was further confirmed by the colocalization between EdeA-GFP and AbpA-RFP, which is a marker of endocytic internalization. It has been established that endocytosis is essential for filamentous growth, and mutations that block endocytosis are lethal or severely debilitating (28, 37). However, the loss of *edeA* leads to reduced growth and hyphal polarity defects but is not lethal, indicating that EdeA is either non-essential or functionally redundant with other genes. Indeed, the FM4-64 assay revealed that the loss of *edeA* significantly reduced the number of endocytosis initiation sites but did not completely block endocytosis. In addition, although *ede1* orthologs in *C. albicans* and *A. fumigatus* are non-essential, *edeA* plays more important roles in pathogenic filamentous fungi than it does in pathogenic yeast (38).

The cell wall protects *A. fumigatus* from external aggression and plays a positive role in its pathogenicity (39, 40). In this study, we demonstrated that EdeA is involved in cell wall biosynthesis in *A. fumigatus*. Multiple lines of evidence implicate that EdeA is crucial for cell wall integrity in *A. fumigatus*. (1) The apical chitin content stained by Calcofluor white is significantly decreased in the Δ*edeA* mutant (2). The Δ*edeA* is more sensitive to cell wall-disrupting reagents than the WT (3). The cell wall of the Δ*edeA* mutant was unusually thick (4). The levels of MpkA phosphorylation were higher in the Δ*edeA* mutant, indicating that the CWI pathway was abnormally activated even under non-stress conditions. The connection between the cell wall and endocytosis is further strengthened by the site mutation assay, which shows that the conserved glutamate within the third EH domain is required for both endocytosis and cell wall integrity. The studies in several fungi have revealed that endocytosis was involved in cell wall integrity. In *A. nidulans*, it has been reported that certain cell wall-modifying enzymes, like ChsB, need to be internalized and recycled through endocytosis and that the loss of *chsB* leads to disruption of rapid apical expansion, resulting in severe damage to hyphal growth (14, 31). In addition, it has been reported that genes involved in endocytosis, such as Pan1p, End3p, and Sla1p, are also required for normal cell wall morphogenesis in *S. cerevisiae*, and the loss of these genes also leads to a thicker cell wall (41, 42). In line with this, it has also been found that there is a phenotypic similarity between the endocytosis-internalizing proteins End3 and the epsin Ent2 in *C. albicans*, both of which exhibit increased sensitivity to cell wall-disrupting reagents after being knocked out (2, 43). Furthermore, the early endocytic patch protein Sla2 and the actin-binding complex Arp2/3 are also involved in the synthesis of the cell wall in *C. albicans* (44, 45). The results of the above research indicate that the loss of cell wall integrity may be a common feature of endocytosis mutants.

Considering the roles of EdeA in endocytosis, hyphal growth, and polarity, EdeA potentially plays a critical role in pathogenicity. Unexpectedly, the pathogenicity assay using the wax moth infection model of *G. mellonella* showed that the Δ*edeA* mutant had increased pathogenicity compared with the WT strain. The cell wall is the major structure that maintains cell morphology and mediates the interactions between *A. fumigatus* and hosts (37). The unexpected increase in pathogenicity in the Δ*edeA* mutant might be caused by its abnormal cell wall morphology, such as the observation of an unusually thick cell wall in the Δ*edeA* mutant. The role of EdeA in pathogenicity needs to be further confirmed and characterized in a mouse infection model.

Structurally, all EdeA orthologs contain three EH domains, indicating the crucial roles of this domain in the function of EdeA homologs. Previous reports have shown that Eps15 and Ede1 primarily bind to interacting proteins through the EH domains and play a role in endocytosis and signal transduction processes (34). Interestingly, all EH domains contain two EF-hand motifs, which have been reported to have the ability to bind to calcium ions (35). Previous studies have shown that the 12th amino acid (glutamate) in each EF-hand loop plays a crucial role in the process of calcium ion binding (46). However, it is unknown whether this site is crucial for the function of EdeA. In this study, we identified that the 348th glutamate within the third EH domain of EdeA, which has the ability to bind calcium, is crucial for its subcellular localization, cell wall integrity, and endocytosis, as determined by site mutation assay. This result also indicates that the binding of calcium might be crucial for the EdeA functions. However, whether EdeA has the ability to bind the calcium ions needs to be further explored.

## MATERIALS AND METHODS

### Strains, media, and culture condition

The strains used in this study are listed in Table 1. The A1160 strain was purchased from the Fungal Genetics Stock Center (FGSC). A1160^C^ (reintroducing *pyr4* into A1160) was used as the parental WT strain (47). *A. fumigatus* strains were grown on minimal medium (MM, containing 1% glucose, trace elements, and 20× salt) and YAG (containing 2% glucose, trace elements, and 0.5% yeast extract), as described previously (48). For fresh conidia collection, *A. fumigatus* strains were grown on YAG for 2 days and collected using 0.02% (vol/vol) Tween 20 in a saline solution.

**TABLE 1:** Strains used in this study

Strain	Genotype and source	Reference
A1160	Δ*ku80*, *pyrG*	FGSC
WT (A1161)	A1160*::pyr4*	(47)
Δ*edeA*	Δ*ku80, pyrG,* Δ*edeA::pyr4*	This study
*edeA^E348A^*	Δ*ku80, pyrG,* Δ*edeA::pyr4, edeA (p):: edeA^E348A^::hph*	This study
*edeA^C^*	Δ*ku80, pyrG,* Δ*edeA::pyr4,edeA::hph*	This study
EdeA-GFP	A1160, *pyr4, edeA::gfp::hph*	This study
EdeA*^E348A^*-GFP	Δ*ku80, pyrG,* Δ*edeA::pyr4, edeA (p):: edeA^E348A^::gfp::hph*	This study
EdeA-GFP/AbpA-RFP	A1160::*pyr4, edeA::gfp::hph, abpA::rfp::pheI*	This study

### Phylogenetic tree construction

The sequences of EdeA orthologs were downloaded from the NCBI (https://www.ncbi.nlm.nih.gov) and FungiDB (http://fungidb.org/fungidb) websites. A phylogenetic tree was constructed using the neighbor-joining method with MEGA 11 software, and sequence alignment was conducted using Cluster W. The phylogenetic tree was enhanced using the iTOL software (https:// itol.embl.de/upload.cgi). Conserved domain analysis of proteins was performed using the Conserved Domain Database website on the NCBI and visualized using IBS (Illustrator for Biological Sequences, http://ibs.biocuckoo.org/dbvisualization.php#).

### Genetic manipulation of *A. fumigatus*

The homologous recombination method was used to construct the *edeA* null mutant with *pyr4* as a marker. First, a flanking sequence of approximately 1.0 kb upstream and downstream of *edeA* was amplified from A1160 genomic DNA using primers EdeA-p1/p3 and EdeA-p2/p6. The *pyr4* gene was amplified from the plasmid pAL5 using the primers *pyr4*-F and *pyr4*-R. Next, the three fragments were fused to create the gene knockout cassette with primers EdeA-p2/p5 (49). Finally, the knockout cassette was transformed into the WT strain A1160 as previously described (47, 50).

To generate the *edeA*-complemented strain *edeA^C^*, the *edeA*-complemented fragments, which include 1.0 kb of the upstream flanking regions and the *edeA* ORF, were amplified with the primer pair Com-*edeA*-F/Com-*edeA*-R. Subsequently, it was cloned into plasmid pAN7-1 at the HindIII cleavage site using the ClonExpress MultiS Single-Step Cloning Kit (Vazyme Biotech, C113-02). Then, the complemented plasmid was transformed into Δ*edeA* to generate the *edeA^C^* strain.

To obtain the *in situ* EdeA-GFP-labeled strain, the flanking fragments of *edeA* (excluding termination codons) were amplified with the primers EdeA-GFP-P1/P3 and EdeA-GFP-P4/P6, respectively. The GFP-*hph* fragment was amplified using the GFP-*hph*-F/R primers. Next, the three PCR products were fused together using the primers EdeA-GFP-P2 and EdeA-GFP-P5. Afterward, the fused PCR fragment was transformed into the WT strain A1160^C^.

For the EdeA-GFP and AbpA-RFP colocalization strain, the flanking fragments of *abpA* (exclude termination codons) were amplified from the WT genome using the primers AbpA-RFP-P1/P3 and AbpA-RFP-P4/P6, respectively. The RFP-*pheI* fragment was amplified with the primers RFP-*pheI*-F/R. Next, the three PCR products were fused together using the primers AbpA-RFP-P2 and AbpA-RFP-P5. Then, the fused PCR fragment was transformed into EdeA-GFP strain.

To obtain the point mutant strains, fragments of *edeA* were cloned using primers EdeA^E348A^-P1/P2 and EdeA^E348A^-P3/P4 from the A1160 genomic DNA. Two pairs of reverse primers were designed at specific points where mutations were needed. Next, the two sequences were fused using primers EdeA^E348A^-P1/P4. And then, the fragment was cloned into pAN7-1 using the ClonExpress MultiS Single-Step Fusion Cloning Kit, and the plasmid was transformed into the Δ*edeA* mutant.

For the EdeA^E348A^-GFP strain, the flanking fragments were amplified from the EdeA-GFP genome using the primers EdeA^E348A^-GFP-F1/R1 and EdeA^E348A^-GFP-F2/R2. Subsequently, the two fragments were fused together using the primers EdeA^E348A^-GFP-F1 and EdeA^E348A^-GFP-R2. Finally, the fused fragment was transformed into a Δ*edeA* mutant. All primers used in this study are listed in Table S1.

### *A. fumigatus* germination and biomass assays

Fresh conidia of related strains (1 × 10^5^ conidia/mL) were inoculated onto glass coverslips in liquid MM and YG media and grown statically at 37°C for 7 h, 8 h, and 9 h. An Olympus Th4-200 fluorescence microscope with a 20× objective (Olympus Corporation, Tokyo, Japan) was used to capture images of each strain. Images were used to measure the germination rate of *A. fumigatus*, and conidia with an emerging germ tube in the form of a drop were scored as germinated (51). The number of germlings in each image was divided by the total number of conidia and germlings in the entire image. For this purpose, three experiments with more than 200 conidia per strain were carried out, each with three replicates.

To compare the biomass of related strains, fresh conidia of WT, Δ*edeA*, and *edeA^C^* (1 × 10^7^ conidia/mL) were inoculated into liquid MM and YG media and grown for 24 h under shaking condition. After adequate drying, the mycelium was collected and weighed.

### Plate assays

To test the growth phenotype and the sensitivity of the indicated strains to cell wall-disrupting reagents, fresh conidia from two microliters (1 × 10^7^ conidia/mL) of the relevant strains were inoculated onto solid MM and MM plus 100 µg/mL CFW, 50 µg/mL CR, 0.5 µg/mL CAS, and 4 µg/mL CAS and cultured at 37°C for 72 h. Subsequently, the phenotypes were observed and imaged. Colony growth diameter and conidia production per unit area (total number of conidia divided by colony area) of the indicated strains were determined in three independent biological experiments. The colony inhibition ratio is obtained by dividing the colony diameter under different cell wall-disrupting reagents (CFW, CR, and CAS) by the colony diameter grown under control condition (MM). Subtract this ratio from 100%.

### Fluorescence microscopy analyses

To analyze the localization of EdeA-GFP, AbpA-RFP, and EdeA^E348A^-GFP, fresh conidia of the related strains were cultured in liquid MM at 37°C for 13 h on glass coverslips. After growth, the medium was removed, and the hyphae were washed three times with PBS buffer. Analysis of the fluorescence intensity of the images with ImageJ to compare the distribution patterns of EdeA-GFP and AbpA-RFP.

To observe the endocytic membrane trafficking ability of relevant strains, fresh conidia of the indicated strains were cultured in liquid MM for 13 h. Subsequently, the hyphae were washed three times with PBS buffer and stained with the fluorescent lipophilic styryl dye 4–64 (FM4-64, membrane-selective fluorescent vital dye that has been used as a marker of endocytosis in live cells) as previously described (52, 53). For short-term loading, hyphae of the indicated strains were loaded with 5 µM FM4-64 for 2 min in the dark on ice. For long-term staining, hyphae of the indicated strains were incubated with 5 µM FM4-64 for 5 min at room temperature in the dark, followed by internalization for 15 min and 2 h. After staining, hyphae were washed three times with fresh YG medium without FM4-64. All images were captured using an Olympus Th4-200 fluorescence microscope with a 63× oil immersion objective (Olympus Corporation, Tokyo, Japan), and the images were compiled using Adobe Photoshop.

Only a small number of endocytic sites are formed after a staining time of 2 min. The rate of endocytosis can be determined from the number. Count 20 hyphae from each strain and calculate the average number of actin patches. When the cultivation period is extended, the endocytosis rate is determined by dividing the intensity of the plasma membrane by the intensity of the vacuoles. Calculate the fluorescence intensity of the plasma membrane and the vacuole membrane using ImageJ software. Then, divide the fluorescence intensity of the plasma membrane by the fluorescence intensity of the vacuole membrane (54).

### TEM analysis of the cell wall

1 × 10^7^ conidia of WT and Δ*edeA* were cultured in liquid MM and underwent shaking at 37°C, 200 rpm for 24 h. After pouring out the culture medium, hyphae were collected by thoroughly rinsing the surface of the hyphae with distilled water. The hyphae were then fixed with electron microscope fixative containing 2.5% glutaraldehyde and 100 mM phosphate (Cat: G1102-100 mL, Servicebio) for 30 min at room temperature in the dark. Then, they were sent to the company (Wuhan Service Biotechnology, China) for the next step without rinsing. Sectioning the hyphae with a glass knife on the ultramicrotome, followed by staining with lead citrate and uranyl acetate. Finally, pictures were taken by TEM (Hitachi TEM system, HT7800). To measure cell wall thickness, more than 10 measurements were taken for each strain. The average thickness of the inner and outer cell wall layers was measured using ImageJ, and the scale bar on the images was used as a reference (55).

### Western blot

1 × 10^7^ conidia were inoculated in 100 mL of liquid MM medium and shaken at 37°C, 200 rpm for 23 h. After that, 300 µg/mL of CR was added for further culture for 1 h. After incubation, the CR was thoroughly rinsed and the hyphae were collected. Protein was extracted using the lysis buffer (containing 0.2 M NaOH and 0.2% β-Mercaptoethanol). Then, 100% trichloroacetic acid was added to precipitate the protein after thorough vortexing. After centrifugation at 4°C, the precipitate was collected and then heated at 95°C for 5 min (56, 57). Western blotting was then performed to detect the phosphorylation level of MpkA using anti-phospho-ERK1-T202/Y204+ERK2-T185/Y187 (1:2,000, ABclonal Technology Co.) as the primary antibody and anti-rabbit (1:5,000, ABclonal Technology Co.) as the secondary antibody. The enhanced ECL Luminescence Detection Kit (Vazyme, E411) was used to detect blots, and images were captured using a Tanon 4200 Chemiluminescence Imaging System. ImageJ software is used to calculate the intensity of the bands. First, the background is removed. Then, the position of the measured band is selected and marked. Finally, the area of the selected position is calculated to obtain the intensity of the band. For the quantitative analysis of the total protein content, SDS-PAGE gels were stained with Coomassie Brilliant Blue to bind proteins for 30 min and then destained with a destaining buffer (10% acetic acid, 5% absolute alcohol, and 85% dH_2_O) until the bands were clear.

### *G. mellonella* pathogenicity assay

The pathogenicity of the indicated strains was determined using the *G. mellonella* model (36). Fresh conidia of WT (A1161, reintroducing *pyr4* into A1160), Δ*edeA*, *edeA^E348A^*, and *edeA^C^* were collected after 2 days of growth on solid YAG medium. Then, 10 µL of 1 × 10^8^ conidia of the indicated strains were injected into *G. mellonella* larvae through the last pair of prolegs. Meanwhile, 10 µL of PBS was used as a control to observe the percentage of deaths due to physical trauma. All injected larvae were incubated at 37°C for 8 days, and the number of deaths was recorded daily. This experiment was repeated three times independently, with 30 larvae per strain injected each time.

## SUPPLEMENTAL MATERIAL

Fig. S1 msphere.00057-24-s0001.pdf *edeA* null mutant construction strategy.

Fig. S2 msphere.00057-24-s0002.pdf Labeling with GFP does not affect the growth of EdeA.

Table S1 msphere.00057-24-s0003.docx Primers used in this study.

ASM does not own the copyrights to Supplemental Material that may be linked to, or accessed through, an article. The authors have granted ASM a non-exclusive, world-wide license to publish the Supplemental Material files. Please contact the corresponding author directly for reuse.
