# Supplementary material for: The EH domain-containing protein, EdeA, is involved in endocytosis, cell wall integrity, and pathogenicity in Aspergillus fumigatus
Source: mSphere. 2024 Apr 30;9(5):e00057-24. doi: 10.1128/msphere.00057-24 (PMC11237632; doi:10.1128/msphere.00057-24)
Supplement: Fig. S1 — edeA null mutant construction strategy. [file msphere.00057-24-s0001.pdf]

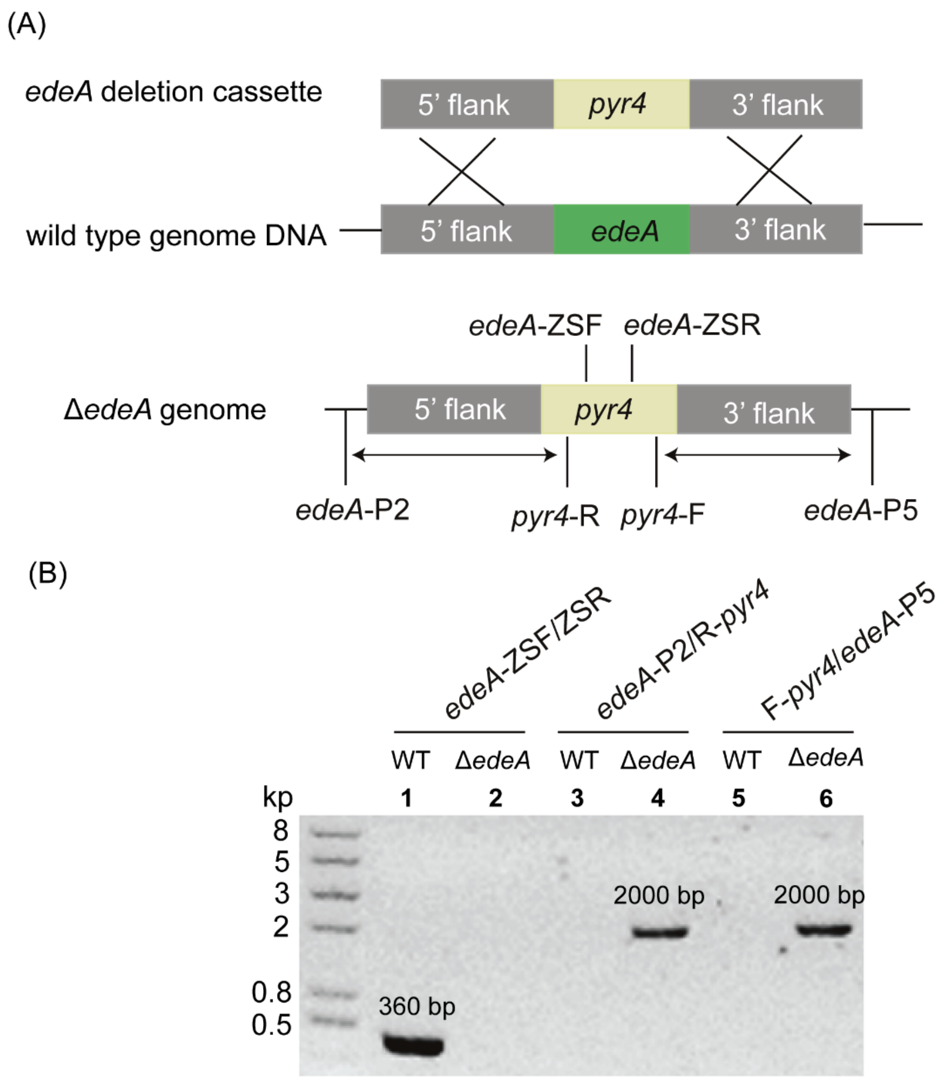

Fig. S1 The *edeA* null mutant construction strategy. (A) Diagram showing the strategy of *edeA* null mutant construction. (B) Diagnostic PCR validation of *edeA* null mutant. The genome of Δ*edeA* were used as the template for PCR with primer pairs *edeA* ORF own pairs *edeA*-ZSF/*edeA*-ZSR (line 1-2), *edeA*-P2/R-*pyr4* (Left arm, line 3-4) and F-*pyr4*/*edeA*-P5 (Right arm, line 5-6), and the wild type genome was used as the control.
