# Supplementary material for: The EH domain-containing protein, EdeA, is involved in endocytosis, cell wall integrity, and pathogenicity in Aspergillus fumigatus
Source: mSphere. 2024 Apr 30;9(5):e00057-24. doi: 10.1128/msphere.00057-24 (PMC11237632; doi:10.1128/msphere.00057-24)
Supplement: Fig. S2 — Labeling with GFP does not affect the growth of EdeA. [file msphere.00057-24-s0002.pdf]

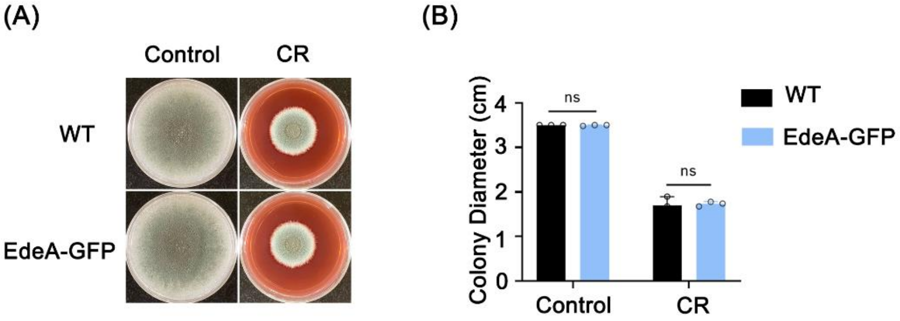

Fig. S2 Labeling with GFP do not affect the function of EdeA. (A) Colony growth of WT and EdeA-GFP strains grown on MM in the presence and absence of cell wall disrupting reagents CR (50 μg/ml) at 37°C for 72 h. (B) Quantitative analysis of colony diameter of indicated strains.
