# Supplementary material for: The EH domain-containing protein, EdeA, is involved in endocytosis, cell wall integrity, and pathogenicity in Aspergillus fumigatus
Source: mSphere. 2024 Apr 30;9(5):e00057-24. doi: 10.1128/msphere.00057-24 (PMC11237632; doi:10.1128/msphere.00057-24)
Supplement: Table S1 — Primers used in this study. [file msphere.00057-24-s0003.docx]

**Table S1. Primers used in this study**

| **Name/Purpose** | **Sequence (5’ to 3’)** |
| --- | --- |
| **Genes deletion and confirmation** | |
| EdeA P1 | GATGGTCAAGTGTGACCCCCGCA |
| EdeA P2 | GCTGCATGCTGTCCAACACC |
| EdeA P3 | CGATTAAGTTGGGTAACGCCAGATTGTAGCTCAGGAGGAGTGC |
| EdeA P4 | AAGTAGCCAGTTCCCGAAAGCATTGACTCTTCTCTAATTTCAG |
| EdeA P5 | CCGCGCCCGCACCGCCCTCG |
| EdeA P6 | TCTCTCCGTCCTCTTCTCAGC |
| EdeA ZSF | GGTCACCGACAATGTGTAGA |
| EdeA ZSR | TGAATTTATTGACGTCTTCC |
| EdeA-GFP P1 | CTGTGACCTCATCTACCAAGC |
| EdeA-GFP P2 | AATGGTACAGCTCCTGCTGC |
| EdeA-GFP P3 | CCAGCGCCTGCACCAGCTCCGCGTCGTTTGCAACCTGAAAG |
| EdeA-GFP P4 | GCTCCTCTTCTTTACTCTGAATTGACTCTTCTCTAATTTC |
| EdeA-GFP P5 | CCGCGCCCGCACCGCCCTCG |
| EdeA-GFP P6 | TCTCTCCGTCCTCTTCTCAGC |
| Com-*edeA*-F | ACTCGACCTGCAGGCATGCAAACGGCAAGTACATGGCCAC |
| Com-*edeA*-R | AAACGACGGCCAGTGCCAAGCTTCGGTTATACTCGAAGAGAA |
| Pyr4F | TGGCGTTACCCAACTTAATCG |
| Pyr4R | GCTTTCGGGAACTGGCTACTTAT |
| Hph SF | AGATCATGGTTGACCGGTGCC |
| Hph SR | CGGAGCATTCACTAGGCAACCAT |
| AbpA-RFP-P1 | TGAATCGTCTGTTGACCAGTC |
| AbpA-RFP-P2 | AGGAATTGACGACTGGACCG |
| AbpA-RFP-P3 | CCAGCGCCTGCACCAGCTCCCTCCTCGAGTTGTACATAGT |
| AbpA-RFP-P4 | GCTTACATTCACGCCCTCCTGTTATGGCTGCTCTTTCTCT |
| AbpA-RFP-P5 | CAGCTTATTCACCGTTATAA |
| AbpA-RFP-P6 | AAATCGACCATGGTAAGATT |
| RFP-*pheI*-F | GGAGCTGGTGCAGGCGCTGG |
| RFP-*pheI*-R | AGGAGGGCGTGAATGTAAGC |
| EdeA^E348A^-P1 | CACTCGACCTGCAGGCATGCAATTCCGAAGAATGGCTGTAG |
| EdeA^E348A^-P2 | ACCGCAAATGCGTCCTTCGTC |
| EdeA^E348A^-P3 | GACGAAGGACGCATTTGCGGT |
| EdeA^E348A^-P4 | AACGACGGCCAGTGCCAAGCTTCGGTTATACTCGAAGAGAATC |
| EdeA^E348A^-GFP-F1 | CACTCGACCTGCAGGCATGCAATTCCGAAGAATGGCTGTAG |
| EdeA^E348A^-GFP-R1 | ACCGCAAATGCGTCCTTCGTC |
| EdeA^E348A^-GFP-F2 | GACGAAGGACGCATTTGCGGT |
| EdeA^E348A^-GFP-R2 | AACGACGGCCAGTGCCAAGCTCGGAGCATTCACTAGGCAAC |
| GFP-*hph*-F | GGAGCTGGTGCAGGCGCTGG |
| GFP-*hph*-R | TCAGAGTAAAGAAGAGGAGC |
